# A comparison between the impacts of lecturing and flipped classrooms in virtual learning on triage nurses’ knowledge and professional capability: an experimental study

**DOI:** 10.1186/s12912-023-01353-2

**Published:** 2023-06-15

**Authors:** Mostafa Javadi, Majid Gheshlaghi, Mostafa Bijani

**Affiliations:** 1 Department of Nursing, School of Nursing and Midwifery, Shahid Sadoughi University of Medical Sciences, Yazd, Iran; 2 Student Research Committee, School of Nursing and Midwifery, Shahid Sadoughi University of Medical Sciences, Yazd, Iran; 3 Department of Medical Surgical Nursing, School of Nursing, Fasa University of Medical Sciences, Fasa, Iran

**Keywords:** Professional capability, Triage, Nurses, Flipped classroom, Virtual learning

## Abstract

**Background:**

The quality of triage in emergency department depends on regular evaluation of triage nurses’ professional capabilities and development of programs to improve them. Flipped classrooms are a new approach to learning which can be employed to improve professional capabilities. The present study aims to compare the impact of lecturing to flipped classrooms in virtual learning on the knowledge and professional capabilities of triage nurses in the emergency departments of the state hospitals of Yazd province in south-western Iran in 2022.

**Methods:**

The present study is an experimental work of research. Seventy-four triage nurses participated in the study. Seventy-four triage nurses were randomly allocated to the two groups, including flipped classrooms (group B) and lecturing (group A). The data collection instruments were an emergency department triage nurses’ professional capability questionnaire and a triage knowledge questionnaire. The collected data were analyzed in SPSS v.22 using independent t-test, chi-squared test, and repeated measures analysis of variance. Level of significance was set at *p* ≥ 0.05.

**Results:**

The participants’ mean age was 33.1 ± 4.3 years. As measured one month after the education, the triage knowledge mean score of the nurses who were educated using the flipped classroom method (9.29 ± 1.73) was higher than that of the nurses who were educated via lecturing (8.45 ± 1.788), and the difference was statistically significant (*p* = 0.001). Also, measured one month after the education, the professional capability mean score of the nurses who were educated using the flipped classroom method (140.27 ± 11.744) was higher than that of the nurses who were educated via lecturing (132.84 ± 10.817), and the difference was statistically significant (*p* = 0.006).

**Conclusion:**

There was a significant difference between the pretest and posttest knowledge and professional capability mean scores of both groups immediately after the education. However, measured one month after the education, the mean and standard deviation of the knowledge and professional capability scores of the triage nurses who had been educated via flipped classrooms were higher than those of the nurses in the lecturing group. Thus, virtual learning using flipped classrooms is more effective than lecturing in improving triage nurses’ knowledge and professional capability in the long run.

## Introduction

As one of the most sensitive areas in hospitals, the emergency department provides patients with immediate medical care and is an integral part of healthcare systems [1]. Overcrowding in the emergency department is a serious issue in all healthcare systems across the world, including Iran [2]. In the 1950s in the United States, triage was suggested as a solution to the problem of overcrowding in emergency departments for the first time [3] Triage is prioritizing patients for receiving care based on the severity of their injuries or illness and giving the best treatment to the largest number of patients in the shortest possible time [4]. The effectiveness of triage depends on the knowledge and skill of the emergency department personnel, including the nurses. Nurses are the main performers of triage in emergency departments and play a primary role in prioritizing the needs of patients whose condition is critical [5]. Triage nurses’ key part in prioritizing the needs of patients who are in life-threatening conditions underscores the importance of evaluating their professional capabilities [6]. Professional capability is a broad concept and varies according to individuals’ field of practice, characteristics, positions, and perspectives [7]. A study by Bijani et al. [8] defines professional capability in triage nurses as possessing not only clinical competence and psychological capabilities, but also commitment to the profession. In other words, professional capability in triage nurses is comprised of clinical competence, psychological empowerment, and professional commitment [8].

In view of the great significance of education in developing professional behaviors, it is essential that nurses’ professional capabilities be enhanced via specialized training. The main goal of nurse education is training capable nurses equipped with sufficient knowledge and skill to give care [9]. In a study by O’Connell [10], the nursing students believed that nurse education should stress development of professional capabilities. Among the different approaches to educating learners, traditional lecturing is the most commonly-used method in universities [11]. The disadvantages of lecturing include one-way transfer of information, the lengthiness of lectures, and learners’ inability to retain information absorbed from lectures for long periods [12, 13]. As with other majors, many nursing educational programs employ lecturing. In this method, learners’ personal capacities are not properly addressed, the role of learners is not significant, and learning is superficial. To overcome these shortcomings, educators have begun to use participatory and active methods of learning [14]. At the beginning of the twenty-first century, researchers found a huge gap between nursing education and nursing practice, leading to calls for a fundamental transformation of nursing education [15]. To that end, Benner and MacKinnon et al. proposed changes in the education system, such as moving away from teaching decontextualized knowledge, better integration of active learning in the classroom. New and innovative instructional strategies must be integrated within nursing education to achieve these goals [15, 16]. Application of modern learning approaches is an effective way to improve triage nurses’ professional capabilities. One of these relatively new approaches is flipped classrooms, whose strength concerns the manner of providing information [17]. The structure of flipped classrooms is based on fundamental changes in the lecture-based method and is a transition toward student-centered learning [12]. According to a study by Dadgari et al., flipped classrooms are helpful in preparing nursing students for self-directed learning and should be used alongside traditional methods of learning [18]. A study by Tan et al. [19] showed that use of flipped classrooms resulted in better knowledge, skills, perception, self-learning, critical thinking, and problem-solving skills in nursing students [20]. According to a study by Herbosa, et al. [21] showed that the use implementation of the flipped classroom in nursing education increases performance and is satisfactorily evaluated by both students and faculty. However, more studies are needed that meet methodological quality standards to consolidate the evidence [19].

As one of the most important parts of every hospital, emergency departments are consistently faced with new challenges and changes in protocols and methods of treatments. Accordingly, it is essential that triage nurses’ knowledge and professional capabilities should be evaluated so that their weaknesses and educational needs can be identified and appropriate measures can be taken to help their professional development and enhance the quality of care provided by them. Triage nurses require continuing education and training, and modern innovative learning methods, including flipped classrooms can contribute to enhancement of nurses’ professional capabilities. Most of the available research on flipped classrooms has targeted nursing students, and the impact of this method of education on nurses, especially triage nurses in emergency departments, has not been studied, creating a gap in research in this area. Also, the most common method of educating nurses in clinical environments in Iran is lecturing, and modern methods of learning, including flipped classrooms, are rarely used, which can be attributed to instructors’ unfamiliarity with modern learning approaches. In view of the spread of COVID-19 and nurses’ heavy workload, especially emergency nurses, virtual learning can prove a proper approach to educating caregivers. The present study was conducted to compare the impact of lecturing to flipped classrooms in virtual learning on the knowledge and professional capabilities of triage nurses in the emergency departments of the state hospitals of Yazd province in southern Iran.

### Objective

The present study aimed to compare the impact of lecturing to flipped classrooms in virtual learning on the knowledge and professional capabilities of triage nurses in emergency departments.

### Research questions

Will there be a difference between the pretest and posttest triage knowledge mean scores of triage nurses who are virtually educated via lecturing and triage nurses who are virtually educated via flipped classrooms?

Will there be a difference between the pretest and posttest professional capability mean scores of triage nurses who are virtually educated via lecturing and triage nurses who are virtually educated via flipped classrooms?

## Methods

### Study design, setting and participants

The present study is an experimental work of research with a pretest–posttest design in 2022. The study population consisted of all the nurses who were in practice in the triage units of the state hospitals of Yazd province in southern Iran, met the inclusion criteria of the study, and were willing to participate. The size of the sample was calculated using the software program G-Power and the findings of a study by Haghdoust et al. [21]. Thus, based on a significance level of 0.05 and test power of 80%, sample size was set at 33 nurses. Considering an attrition rate of 10%, the researchers assigned 37 nurses to each group. In total, 74 triage nurses were recruited.

$$n=\frac{\left(Z_\frac\alpha2+Z_\beta\right)^2{2s}^2}{\left({\overline x}_1-{\overline x}_2\right)^2}$$

**Fig. 1:**
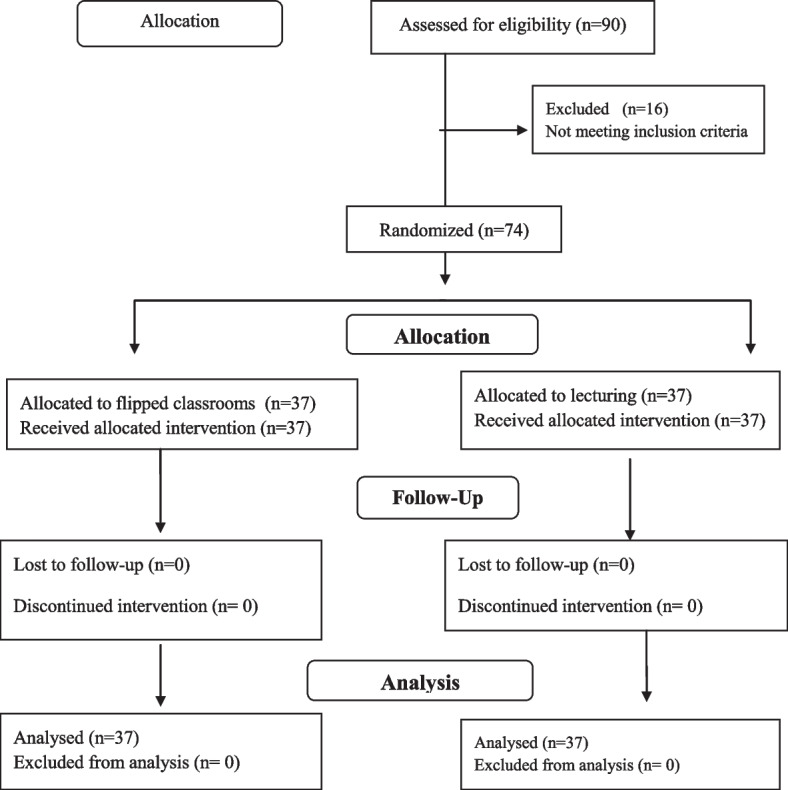
Flow diagram of the participant

The inclusion criteria were: being willing to participate in the study, having at least one year’s experience of practice in a triage unit, being a triage nurse at the time of the study, having access to a computer or smart phone to attend the classes, not having attended a similar training course in the past, and having a relevant bachelor’s degree. The nurses who had attended a similar training course in the past year, had been transferred from the emergency department to another hospital unit, missed more than two sessions of the classes, or failed to fully complete the questionnaires were excluded from the study. The researcher first invited 90 triage nurses to participate in convenience sampling. Of them, 16 triage nurses or did not meet the inclusion criteria were excluded. Therefore, the remaining 74 triage nurses were randomly allocated to the two groups, including flipped classrooms (group B) and lecturing (group A). Thereafter, 74 cards were prepared, including 37 cards labeled A and 37 cards labeled B. These 77 cards were then put in an envelope, and each nurse was asked to draw out one card randomly. Each card labeled A, and B was the lecturing and flipped classrooms. Figure 1 presents the flow diagram of the participants throughout the study (Fig. 1).

Initially, an informed consent form and the questionnaires of the study, i.e. a demographics survey, an emergency department triage nurses’ professional capability questionnaire, and a triage knowledge questionnaire, were submitted to the participants online. The participants were informed that they would be assigned to one of the two study groups.

The virtual education was provided using an Iranian online learning platform named SKYROOM. The content of the education was confirmed by a number of nursing professors and emergency medicine experts. Designed for six 90-min sessions, the education consisted of an introduction to ESI triage algorithm and manner of patient stratification, identification of high-risk emergencies, including respiratory emergencies, trauma, shock, bleeding, cardiac emergencies, poisoning, and endocrine emergencies, professional ethics, management of emotions, resilience, and teamwork skills.

The nurses in the control group attended online lecture-based classes. The experimental group was educated via flipped classrooms: the educational content was made available to the participants 1 week before each virtual session and the nurses were asked to study the content of the files. The educational content, which had already been prepared, consisted of podcasts, PowerPoint slides with narration, and educational videos. The same educational content was used for the virtual flipped classrooms and the virtual classes which used lecturing. However, in the flipped classrooms, the nurses were presented with a summary of the educational content which they had been provided with and most of the class time was dedicated to group discussions and answering the learners’ queries. One week, and again one month after the classes, the questionnaires were completed by the participants again online.

### Pre-class activities for the flipped classroom

At this stage, a video or text was prepared by the teachers and made available to the learners out of class hours. One week prior to presenting each class topic, the teachers made a PowerPoint with a voiceover of the educational content, as well as podcasts and short educational videos, and made them available to the nurses via WhatsApp. Each topic was presented as electronic content lasting 90 min, complying with the standards for electronic content, created by the software Storyline. Each section consisted of a subject, general and specific objectives, a text, a summary, and a final test on the presented educational content.

The participants attended the classes virtually on the online learning platform for content sharing was the interactive Learning Management Systems (LMS) of Shahid Sadoughi University of Medical Sciences of Yazd accessible at https://ssunavid.vums.ac.ir. The software programs allow for managing registrations, follow-ups, evaluations, presentation of plans, and interactions between the learners and teachers. In addition, the educational content uploaded to the servers is easily accessible by laptop, computer, and cellphone. Via the platform, the participants had access to the educational content, assignments, self-evaluations, discussions, and messages. The platform was easy to use and provided guidelines on how to use it in the format of videos and PDF files. Every week, the nurses were provided with an educational module to study and analyze. The nurses were asked to study and reflect on the content individually to practice independent thinking. One day prior to each session, the nurses were given an electronic multiple-choice test to assess their preparation and assure that they have studied the educational content related to that session. A group on WhatsApp was created and all the participants and teachers were invited to join it so that the nurses could be sent notifications and the teachers could answer their queries and monitor the outside-class discussions. All arrangements for each session were made on WhatsApp.

### In-class activities for the flipped classroom

This stage was executed as an online class where the learners asked and answered questions about and discussed the topic of that session for about 90 min. After receiving and checking the required educational content, the nurses attended online classes on an Iranian platform for virtual education, named Skyroom, by following the class links at the specified hours. In class, the learners spent time doing such activities as solving problems raised by the teacher and group discussions. The teacher presented the topic of each session as a clinical scenario and had the learners discuss it. Most of the participants were randomly asked questions about the topic of each session to assess their learning and all the participants had to take in-class quizzes. The nurses’ responses to each question were analyzed in groups. The nurses made comments by typing in the chat box or enabling their microphones and speaking. The teacher guided and monitored the discussions. In addition, during each session, the coordinator of every group shared a summary of the content of that session in the form of a 5-min audio file or a text or a combination of both which had been prepared beforehand in the conference room. The other participants read the summaries and gave feedback. The first author checked the participants’ feedback every day and encouraged the participants to participate further. At the end of each class, the teacher corrected the learners’ misconceptions, provided feedback and a summary, and highlighted the key points in the content and the clinical scenarios.

### Post-class activities

Extended tasks (descriptive and problem-based) were assigned after the educational content of each session had been covered to reinforce learning. The assignments were uploaded to the assignment section of the platform and checked by the second author, who gave feedback within a certain period of time (about 2 weeks).

### Teaching methods in the control group

The nurses in the control group attended online lecture-based classes.

### The data collection instruments

#### The emergency department triage nurses’ professional capability questionnaire

This questionnaire was designed and validated by Bijani et al. in 2018 as a nursing PhD thesis. This self-report questionnaire consists of 35 items which address three areas: clinical competence (items 1 to 20), psychological empowerment (items 21 to 26), and professional commitment (items 27 to 35). The items are scored on a 5-point Likert scale, ranging from very much (5 points) to very little (1 point). The respondents’ total score is the sum of their scores for each item. The score range is between 35 and 175: a score of 35 to 81 indicates poor professional capability, 82 to 128 indicates average professional capability, and 129 to 175 indicates satisfactory professional capability. Also, each dimension of the questionnaire can be scored separately as follows: clinical competence (1 to 100), psychological empowerment (1 to 30), and professional commitment (1 to 45). The internal consistency (Cronbach’s alpha) of the entire scale was 0.89; the internal consistency of each dimension was found to be 0.92 for clinical competence, 0.87 for psychological empowerment, and 0.89 for professional commitment. The total intra- class correlation coefficient (ICC) of the scale was 0.90 [22]. In the present study, the reliability of the questionnaire was assessed using the test–retest method. In doing so, the questionnaire was given to 100 triage nurses in two stages with a 2-week interval. The internal consistency reliability of the questionnaire (Cronbach’s alpha) was 0.97 for the whole scale and 0.96 for clinical competence, 0.95 for psychological empowerment, and 0.89 for professional commitment domains.

#### The triage knowledge questionnaire

To measure the participants’ triage knowledge, the researchers used a researcher-made triage knowledge questionnaire. This questionnaire consisted of 15 multiple-choice questions presented as clinical scenarios. Each correct answer earned one point. The score range was between 0 and 15, with higher scores indicating better triage knowledge. The validity (face and content validity) of the questionnaire was assessed and verified by a panel of experts (consisting of 4 nursing faculty members with experience of triage and 6 emergency medicine experts). The knowledge questions were designed as clinical scenarios in a multiple-choice format. They consisted of questions on triage knowledge of prioritizing patients based on Emergency Severity Index (ESI) triage algorithm and identifying high-risk and life-threatening emergencies, e.g. airway obstruction, bleeding and shock, trauma, burns, cardiac arrest, and poisoning, neurological emergencies, e.g. head and spine cord injuries, brain hemorrhages, seizure, and stroke, and metabolic emergencies, e.g. diabetic ketoacidosis, hypoglycemia and hyperglycemia, etc.

The face and content validity were used to assess the validity of the questionnaire. 15 experts (10 triage nurses and 5 emergency medicine specialists) were interviewed face-to-face and the difficulty level, relevance, and ambiguity were assessed to measure the qualitative face validity of the questionnaire. The quantitative face validity of the questionnaire was explored using impact score. In this regard, impact scores > 1.5 represented the appropriateness of the items [23]. According to the impact scores of all questionnaire items were higher than 1.5. In order to investigate content validity, Content Validity Ratio (CVR), and Content Validity Index (CVI) were used. The necessity of the items was determined by the experts as ‘necessary’, ‘useful but not necessary’, and ‘not necessary’ considering CVR. In doing so, we collected 15 experts (10nursing faculty members with experience in triage and 5 emergency medicine specialists) opinions and values greater than 0.49 were considered acceptable based on the Lawshe table [23]. The CVR of 15 items of was higher than 0.49. Regarding CVI, the 15 experts (10nursing faculty members with experience in triage and 5 emergency medicine specialists) were requested to evaluate the items in terms of relevance, clarity, and simplicity. In this respect, scores above 0.79 were considered acceptable. Based on the results the CVI of 15 items of was higher than 0.79. The Scale-level content validity index/Average (S-CVI/Ave) of the questionnaire was found to be 0.96. Finally, the reliability of the questionnaire was assessed using the test–retest method. In doing so, the questionnaire was given to 100 triage nurses in two stages with a 2-week interval. The intra-class correlation coefficient (ICC) across the 15 item was 0.89, which indicates the appropriate internal consistency of this questionnaire. In the present study, to measure the test–retest reliability of the instrument, the researchers had 50 triage nurses from hospitals outside the research setting complete the questionnaire twice with a 2-week interval. The ICC of the questionnaire was found to be 0.95.

What follows is an example of a clinical scenario on the triage knowledge questionnaire:

A 10-year-old child with severe drowsiness and fatigue is in the triage unit. After examining the patient, the nurse observes that the patients’ pupils are contracted and pinpoint. Pulse oximetry shows oxygen saturation to be 90%. Which triage category should the nurse assign the patient to?

A) Category 1
B) Category 2
C) Category 3
D) Category 4

The questionnaires (triage nurses’ professional capability questionnaire and triage knowledge questionnaire) were completed by triage nurses in two groups before, after, and one month after the intervention.

### Statistical analysis

The collected data were analyzed in SPSS v.22. For all the statistical tests, considering a 95% confidence interval, *p*-values of smaller than 0.05 were statistically significant. According to the Kolmogorov–Smirnov normality test research variables had a normal distribution. Independent t-test was used to measure the difference between the quantitative demographic variables of the two study groups, and chi-square test was used to determine the difference between the qualitative variables of the two study groups. The researchers used repeated measures ANOVA to study the differences between the two groups across the various dimensions of the professional capability and triage knowledge questionnaires.

### Ethical considerations

The present study was conducted by the principles of the revised Declaration of Helsinki, a statement of ethical principles that direct physicians and other participants in medical research involving human subjects. All participants signed the informed consent to participate in the study. All personal information would remain confidential, and that they were free to withdraw at any stage of the study. The participants were assured of the anonymity and confidentiality of their information. Moreover; the local Ethics Committee approved the study of, Research Ethics Committees of School of Medicine- Shahid Sadoughi University of Medical Sciences, Shahid Sadoughi, Iran (Ethical code: IR.SSU.MEDICINE.REC.1400. 177).

## Results

In total, 74 triage nurses participated in the study: 40 (54%) of the nurses were male and 63 (85%) had a bachelor’s degree. The average age of the participants in the flipped classroom and lecturing groups were 33.15 ± 4.361 and 33.12 ± 5.401 years respectively and there was not a significant difference between the means of the two groups’ ages. Independent t-test and chi-square test shows there was no significant difference in demographic variables among the flipped classroom and lecturing groups (Table 1).

**Table 1:** Comparison of the triage nurses demographic characteristics between two groups

Groups		Flipped classroom		Lecturing		*P* -value
Variable		N	%	N	%	
Gender	Female	**18**	48.6	16	43.2	0.816^*^
	Male	**19**	51.4	21	58.8	
Education	Bachelor’s	**32**	85.5	32	85.5	0.987^*^
	Master’s	**5**	13.5	5	13.5	
Age	Mean ± SD			Mean ± SD		0.979^**^
	33.15 ± 4.361			33.12 ± 5.401		
Work experience	7.62 ± 4.286			7.88 ± 5.351		0.796^**^

^*^Chi-square test

^**^Independent sample t-test

The results of the independent t-test showed that there were no statistically significant differences triage knowledge mean scores between the two study groups in the pretest stage. However, after the intervention, the triage knowledge mean score of the nurses in the flipped classroom was higher than that of the nurses who were educated via lecturing, and the difference was statistically significant (*p* = 0.001). Moreover, measured one month after the completion of the intervention, the difference in means of the triage knowledge between the two groups was significant (*p* = 0.066) (Tables 2 and 3).

**Table 2:** A comparison between the two groups’ triage knowledge mean scores as measured before, immediately after, and one month after the education

Stage	Before the education M ± SD	immediately after the education M ± SD	one month after the education M ± SD	F	*P* -value (repeated measures ANOVA)
Group					
Flipped classroom	7.48 ± 1.95	9.29 ± 1.73	9.21 ± 1.70	33.21	0.001
Lecturing	7.38 ± 1.55	8.216 ± 1.75	8.17 ± 1.78	10.47	0.001
T	0.26	2.672	1.86		
*P* –value (independent t-test)	0.793	0.001	0.066

**Table 3:** Comparison of the difference in means of the triage knowledge scores as measured before, immediately after, and one month after education in two groups

Stage	Before and immediately after education		Before and one month after education		Immediately after and one month after education	
Group	difference in means	*P* –value	difference in means	*P* –value	difference in means	*P* -value
Flipped classroom	1.811	0.001	1.730	0.001	0.081	0.989
Lecturing	0.838	0.001	1.081	0.001	0.243	0.964

The results of the study showed that the difference between the pretest and posttest professional capability mean scores of the two groups was not statistically significant. However, measured one month after the intervention, the professional capability mean score of the nurses who were educated using the flipped classroom method (140.27 ± 11.744) was higher than that of the nurses who were virtually educated via lecturing (132.84 ± 10.817), and the difference in means of the professional capability scores was statistically significant (*p* = 0.006) (Tables 4 and 5). Moreover, the results of the test question item analysis report in two groups showed that the difficulty and discrimination index of the triage knowledge questions test it has been at an acceptable level (Table 6).

**Table 4:** A comparison between the two groups’ professional capability mean scores as measured before, immediately after, and one month after the education

Stage	Before the education M ± SD	immediately after the education M ± SD	one month after the education M ± SD	F	*P* -value (repeated measures ANOVA)
Group					
Flipped classroom	129.89 ± 14.824	136.68 ± 14.277	140.27 ± 11.744	36.86	0.001
Lecturing	128.41 ± 14.380	132.24 ± 13.115	132.84 ± 10.817	10.119	0.001
T	0.438	1.391	2.831		
*P* –value (independent t-test)	0.663	0.169	0.006

**Table 5:** Comparison of the difference in means of the professional capability scores as measured before, immediately after, and one month after education in two groups

Stage	Before and immediately after education		Before and one month after education		Immediately after and one month after education	
Group	difference in means	*P* -value	difference in means	*P* -value	difference in means	*P* -value
Flipped classroom	1.324	0.001	2.405	0.001	1.081	0.168
Lecturing	1.162	0.012	1.432	0.124	0.270	0.976

**Table 6:** Difficulty and discrimination index of the triage knowledge questions test in two groups

Item analysis	Before the intervention	Immediately after the intervention	One month after the intervention
Difficulty index	0.49	0.62	0.74
Discrimination index	0.37	0.49	0.54

## Discussion

The present study was conducted to compare the impact of lecturing to flipped classrooms in virtual learning on the knowledge and professional capability of triage nurses in the emergency departments of the state hospitals of Yazd province in south-western Iran. The findings of the study showed that, after the education intervention, the triage knowledge mean score of the flipped classroom group was higher than that of the lecturing group. The results of a U.S. study by Fatima et al. [24] showed that flipped classrooms are an effective method to increase learner participation and encourage in-depth and active learning, which is consistent with the findings of the study [24]. However, another study conducted in the U.S. investigated the impact of different learning methods on ophthalmology interns’ ability to cope with the challenges in their training and concluded that there was no significant difference between lecturing and flipped classrooms in affecting the learners’ final grades [25]. The discrepancy between these findings may be due to the great importance of grades to students; therefore, the students in both groups tried their best to absorb the educational content. In flipped classrooms, students learn materials more deeply and feel a greater sense of responsibility for their learning. Video lectures allow for more flexibility in learning and give learners the chance to review previous sessions. Another reason for better learning in flipped classrooms is the fact that it allows learners to learn at their own pace: some learners need to listen to a lecture or read a text several times before they absorb it, while others learn what there is to learn the first time.

In the present study, there was not a statistically significant difference between the pretest and posttest knowledge mean scores of the flipped classroom methods and lecturing. However, measured one month after the education intervention, the triage knowledge mean score of the flipped classrooms was significantly higher than that of the lecturing. The results showed that both the lecturing and flipped classroom methods transferred knowledge to the learners, but education via flipped classrooms resulted in longer retention of knowledge by the learners. One of the shortcomings of lecturing was the learners’ failure to retain the knowledge transferred to them for long periods, which was consistent with the results of a study by Sadeghi et al. In their study, Sadeghi et al. found that lectures resulted in one-way transfer of information, took a long time to present, and did not guarantee long-term knowledge retention: the learners forgot 80% of the material presented to them in 8 weeks [26]. A study in Iran compared the impact of learning via lecturing to a combination of virtual learning and lecturing on college students’ level of knowledge and found that the blended approach resulted in higher levels of knowledge and greater satisfaction in the students [27]. Similarly, several other studies reported better outcomes for blended and learner-centered methods of learning than the conventional method of lecturing [28, 29]. Comparing the effects of the three approaches of online learning, face-to-face learning, and blended learning on medical students, Bahadorani et al. found that blended learning resulted in higher knowledge and skill mean scores on the part of the students than the other methods [30]. At the same time, a few other studies reported that blended methods of learning and lecturing were equally effective, which is not consistent with the findings of the present study [31, 32]. This discrepancy may be due to the fact that in the aforementioned studies, the subjects were tested a short time after their education; therefore, the difference between the blended learning group and lecturing group was not significant. The results of the present study showed that, in the long term, flipped classrooms result in better retention of knowledge by the learners. Longer knowledge retention in virtual learning, including flipped classrooms, can be attributed to the active and student-centered nature of this approach. In addition, learners vary in their learning potential: some learners need to review a lesson several times before they can commit it to memory, a condition available in flipped classrooms, which explains the greater efficacy of virtual learning, especially flipped classrooms [33]. Similarly, a study by Lelean & Edwards [34] showed that the implementing the flipped classroom, which included improved understanding and retention of knowledge. This was mainly due to students being able to watch the pre-class videos until they understood the content, and before applying this new knowledge to case studies and activities in class [34].

Comparing the impact of flipped classrooms to that of lecturing on nursing students’ knowledge of medical equipment, Mirdehghan et al. [33] found an increase in the mean scores of both groups of students, but the difference between the pretest and posttest knowledge mean scores of the students in flipped classrooms was greater. Thus, instructors at nursing schools are recommended to use flipped classrooms to educate students in using medical equipment [33]. Similarly, a study by AL-Mugheed and Bayraktar [35] showed that the venous thromboembolism (DVT) knowledge mean score of the nursing students in flipped classrooms was higher than that of the students who were educated via lecturing. Also, most of the students in flipped classrooms reported that learning in flipped classrooms increased their motivation for learning, improved their comprehension of the course content, and enhanced their communication and clinical thinking skills [35]. The findings of a study by Taskin and Bahar (2021) showed that the knowledge and skill mean scores of the nursing students for measuring vital signs were greater in flipped classrooms as compared to verbal-based traditional education [36]. On a similar note, Chaudhuri et al. (2022) found that, compared to lecturing, flipped classrooms resulted in better basic life support (BLS) knowledge and skill mean scores in medical students [37].

The findings of the present study showed that the posttest professional capability mean score of the flipped classroom group was higher than that of the lecturing group. Similarly, a study by Gopalan and Klann [38] reported that the students who took their psychology course in flipped classrooms felt significantly more prepared for their tests than the students who were educated via the conventional approach. In a meta-analysis conducted by Oliver et al. 2020 [39], a blend of learning models in flipped classrooms for nursing students were reviewed. The results showed that effective flipped classroom models enhanced participatory learning, teamwork, and active learning and improved the students’ critical thinking and decision making skills for better patient care [40]. Another study in China evaluated the effectives of different approaches to training medical students in conducting electrocardiogram and found that flipped classrooms increased the learners’ interest in learning and improved their self-learning abilities [41]. The reasons for better learning and learner participation in flipped classrooms include the relevance of the educational content to the learners’ lives and learners’ freedom to participate in and control their learning environment in this learner-centered approach, factors which are important to adult learners according to the adult learner theory. Since emergency departments, as one of the most important units in every hospital, are constantly faced with new challenges and changes in protocols and treatments, emergency room nurses need continuous education and training. The important role of innovative approaches to learning in preparing nursing graduates and practicing nurses in all hospital units, especially the emergency room, and the findings of studies, the present study included, verifying the benefits of flipped classrooms lead the researchers to conclude that flipped classroom models are an effective method of education in nursing.

### Limitations

In the present study, since the subjects were practicing in the same department, it was possible that the two groups shared their learning in their interactions. However, the fact that the subjects had been randomly divided into two groups minimized the impact of confounding variables. Also, to eliminate the Hawthorne Effect, while the experimental group was being educated, classes on a different topic were held for the control group. Yet, it was not possible for the researchers to completely eliminate the Hawthorne Effect.

### Strengths

The present study is the first interventional attempt at comparing the impact of lecturing to flipped classrooms in virtual learning on the knowledge and professional capability of triage nurses in south-western Iran, which makes it an innovative work. Another the strength of the study is the researchers’ use of specialized questionnaires for assessment of triage nurses’ professional knowledge and capability.

## Conclusion

The findings of the present study showed a significant increase in the knowledge and professional capability mean scores of the triage nurses in both groups immediately after the intervention. However, measured one month after the intervention, the increase was significant only in the case of the flipped classroom group, which shows that, in the long term, flipped classrooms were more effective than lecturing in increasing triage nurses’ knowledge and professional capability. Therefore, flipped classrooms are recommended as an effective and important teaching strategy in clinical nursing education and nursing students.

## Data Availability

The datasets generated and/or analysed during the current study are not publicly available due to the necessity to ensure participant confidentiality policies and laws of the country but are available from the corresponding author on reasonable request.

## Abbreviations

ICC: Intra-class -correlation coefficient
S-CVI /Ave: Scale-level content validity index/ Average
